# *N*-Acetylcysteine as a Potential Antidote and Biomonitoring Agent of Methylmercury Exposure

**DOI:** 10.1289/ehp.10383

**Published:** 2007-10-17

**Authors:** David A. Aremu, Michael S. Madejczyk, Nazzareno Ballatori

**Affiliations:** Department of Environmental Medicine, University of Rochester School of Medicine, Rochester, New York, USA

**Keywords:** *N*-acetylcysteine, antidote, biomarker, biomonitoring, embryotoxicity, methylmercury, toxicity

## Abstract

**Background:**

Many people, by means of consumption of seafood or other anthropogenic sources, are exposed to levels of methylmercury (MeHg) that are generally considered to be quite low, but that may nevertheless produce irreversible brain damage, particularly in unborn babies. The only way to prevent or ameliorate MeHg toxicity is to enhance its elimination from the body.

**Objectives:**

Using *N*-acetylcysteine (NAC), we aimed to devise a monitoring protocol for early detection of acute exposure or relatively low MeHg levels in a rodent model, and to test whether NAC reduces MeHg levels in the developing embryo.

**Results:**

NAC produced a transient, dose-dependent acceleration of urinary MeHg excretion in rats of both sexes. Approximately 5% of various MeHg doses was excreted in urine 2 hr after injection of 1 mmol/kg NAC. In pregnant rats, NAC markedly reduced the body burden of MeHg, particularly in target tissues such as brain, placenta, and fetus. In contrast, NAC had no significant effect on urinary MeHg excretion in preweanling rats.

**Conclusions:**

Because NAC causes a transient increase in urinary excretion of MeHg that is proportional to the body burden, it is promising as a biomonitoring agent for MeHg in adult animals. In view of this and because NAC is effective at enhancing MeHg excretion when given either orally or intravenously, can decrease brain and fetal levels of MeHg, has minimal side effects, and is widely available in clinical settings, NAC should be evaluated as a potential antidote and biomonitoring agent in humans.

Methylmercury (MeHg) ranks among the most highly bioconcentrated toxic metals in the human food chain. It is typically bio-magnified in fish at the top of the food chain up to 100,000 times the concentration in surrounding waters, making fish consumption the single major source of human MeHg exposure [Agency for Toxic Substances and Disease Registry (ATSDR) 1992; Counter and Buchanan 2004; Gonzalez et al. 2005; Horvat et al. 2003]. MeHg is also readily absorbed by inhalation and dermal contact, and it is able to cross the blood–brain and blood–placental barriers, causing irreversible damage to the brain. According to a National Academy of Sciences report (NAS 2000), 60,000 fetuses are at risk of MeHg-induced brain damage in the United States, to such a degree that it may affect the children’s school performance.

In contrast to the rarity of clinical MeHg toxicity, many people are exposed to MeHg levels that, while generally considered to be quite low, may produce subtle neurologic effects, particularly in infants and children (Chapman and Chan 2000; Clarkson 1998; Counter and Buchanan 2004). MeHg toxicity exhibits a latent period after exposure such that by the time clinical signs and symptoms have appeared, it is usually too late to reverse the damage (Clarkson 1997). To assess MeHg exposure and/or toxicity, several biomarkers have been proposed (Ali et al. 1992; Diaz et al. 2004; Iyengar and Rapp 2001; Thompson et al. 1999). The sampling techniques for many of these biomarkers are invasive and therefore unrealistic for use in humans for preventive intervention. Hair remains the current medium of choice for assessing MeHg exposure in humans (Clarkson 1998). The total or segmental hair level of MeHg provides an excellent measure of exposure history over a recent defined past, which may span several months, because the growth rate of hair is about 1 cm/month. However, hair analysis is not useful for acute exposures or for the assessment of current body burden (Boischio and Cernichiari 1998; Boischio et al. 2000; Cox et al. 1995). Furthermore, the new U.S. Environmental Protection Agency (EPA) reference level of only 1–2 ppm (Rice et al. 2000) is also close to the background level found in hair. These new U.S. EPA guidelines also increase the number of people that are considered at risk of MeHg poisoning, and thus they increase the number of people that need to be monitored both in epidemiologic studies and in the general population.

Several chelating agents have been studied as potential MeHg antidotes and more recently as a provocative mercury “challenge” for the purpose of biomonitoring (Aposhian et al. 1995; Domingo 1995; Frumkin et al. 2001; Risher and Amler 2005). Unfortunately, all chelating agents identified so far have significant side effects and are also known to differ in their efficacy for various forms of mercury, route of administration, and route of excretion (Risher and Amler 2005). The current choices and the most widely used MeHg chelators are the thiol-containing compounds *meso*-2,3-dimercaptosuccinic acid (DMSA, succimer, captomer, chemet) and 2,3-dimercapto-1-propanesulfonate (DMPS, dimaval, unithiol) (Risher and Amler 2005). However, DMSA and DMPS have limited stability in solution, limited availability for human use, and a propensity to mobilize other minerals (especially divalent cations) essential for normal physiologic functions (Grandjean et al. 1997; Mann and Travers 1991; Nogueira et al. 2003; Risher and Amler 2005). *N*-Acetylcysteine (NAC) has also been shown to be remarkably effective at enhancing MeHg excretion in mice (Ballatori et al. 1998). Mice that received NAC in the drinking water (10 mg/mL) starting 48 hr after MeHg administration excreted 47–54% of the mercury in urine over the subsequent 48 hr, compared with only 4–10% in control animals (Ballatori et al. 1998). NAC is a relatively simple, nontoxic *N*-acetyl derivative of cysteine, which contains a thiol group that is stabilized by acetylation of the amino group. Unlike other chelating agents, NAC is a potent antioxidant/detoxicant and does not alter tissue distribution of essential metals (Hjortso et al. 1990). NAC was previously shown to be protective against MeHg-induced embryo-toxicity (Ornaghi et al. 1993), although the mechanism was not identified. Thus, these findings open the possibility that NAC may be used to accelerate MeHg excretion and thus minimize its toxicity.

In the present study we tested the hypothesis that a standardized dose of NAC will produce a transient increase in urinary MeHg excretion that is proportional to the body burden of MeHg using a rodent model. We also examined whether NAC is effective at accelerating urinary excretion of MeHg in rats of both sexes and at different ages, and whether it can diminish MeHg levels in the developing embryo. Because the toxic effects of MeHg often do not manifest themselves for several days or even weeks after exposure and because the effects are largely irreversible once they appear (Clarkson 1997), early detection of exposure and prompt therapeutic intervention with a complexing agent, such as NAC, is critical for preventing or minimizing toxicity.

## Materials and Methods

### Animals and reagents

Wistar rats were obtained from Charles River Laboratories (Kingston, NY). They were allowed an acclimatization period of at least 5 days in a temperature- and humidity-controlled room with a 12-hr alternating light cycle, and were maintained on standard laboratory chow with water *ad libitum*. Animals were used for experiments at 250–300 g body weight and with four animals per group, except where otherwise stated. All experiments were conducted in accordance with the guidelines of the National Institute of Health for care of laboratory animals [Office of Laboratory Animal Welfare (OLAW) 2002]. We obtained [^14^C]MeHg from American Radiolabeled Chemical, Inc. (St. Louis, MO), NAC from Sigma Chemical Co. (St. Louis, MO), and other chemicals and reagents from J.T. Baker (Philipsburg, NJ) and VWR (West Chester, PA).

### Surgical procedure and urine collection from anesthetized rats

The animals were treated humanely and with regard for alleviation of suffering. Rats were anesthetized by intraperitoneal administration of pentobarbital sodium (55–60 mg/kg). The right jugular vein was exposed, a nick was made in the vein, and a PE-50 tube (Becton Dickinson & Co., Sparks, MD) was inserted into the vein and tied in place by a ligature at the distal end of the vein. The PE-50 tube was filled with glucose solution (140 mM) via a 22-gauge needle connected to a 20 mL syringe using a syringe pump (model 341B; Sage Instruments, Boston, MA); an infusion rate of 4.1 mL/hr was applied throughout the experiments. The trachea was cannulated using a PE-205 tube to allow easy passage of air. A rectal probe and heating lamp connected to a Tele-Thermometer (Yellow Springs Instrument, Yellow Springs, OH) were used to monitor and maintain the rat’s body temperature at 37°C. Following laparotomy, the urinary bladder was cannulated using the flared end of a PE-50 tube that was tied in place. Excreted urine was collected at 30-min intervals into tared 12 × 75 mm test tubes throughout the experiment. [^14^C]MeHg (0.1 μmol/kg, except when otherwise stated) was given intravenously (iv) at a rate of 200 μL/min after the collection of the first urine sample. Two hours after the injection of [^14^C]MeHg, a bolus dose of NAC (1.0 mmol/kg, except when otherwise stated) was given iv at a rate of 200 μL/min. In another experiment, a second NAC dose was given 1 hr after the first dose. The experiment was terminated 2.5 hr after NAC injection. At the end of the experiments, we collected 1.0 mL blood by cardiac puncture and then removed and weighed the liver, kidney, spleen, and brain.

### MeHg disposition in pregnant rats

Rats were placed individually in stainless-steel metabolic cages (Lab Products Inc., Rochelle Park, NJ) and were allowed to acclimate to the cages for 3 days. On gestation day (GD) 14, rats were injected iv via lateral tail vein with [^14^C]MeHg (2 mL/kg of 0.1 μmol MeHg/kg with 5 μCi ^14^C/kg); after 24 hr, some rats were supplied drinking water containing 10 mg/mL NAC *ad libitum* for 2 days, while others served as controls. Fresh NAC solution was prepared daily. At the end of the experiments, the uterus was removed and two fetoplacental units were selected from each animal. The fetus and placenta were separated, and other maternal tissues were removed and weighed.

### Determination of [^14^C]MeHg content

Aliquots of 100 μL were taken from all urine samples (on exceptional cases, < 100 μL but at least 50 μL of urine was used). Opti-Fluor (5 mL; Packard Instrument Co., Meriden, CT) was added to urine aliquots in scintillation vials, and the mixture was vortexed and allowed to stand in the dark overnight before counting on a Beckman 6500 scintillation spectrometer (Beckman Coulter, Inc., Fullerton, CA). A small cross section (~ 0.2 g) cut from each tissue or 1.0 mL blood was put into a tared 20-mL glass vial and stored at −20°C for not more than 1 week before further treatment. To minimize blood content in the tissue samples, the tissues were blotted on absorbent paper. In the case of the brain, we carefully removed the meninges, along with small blood clots, under a light microscope. To solubilize the tissues, we added 1.0 mL Solvable (Packard Instrument Co.) per 0.1 g tissue or 0.5 mL blood; the glass vial was heated in a shaking incubator at 60°C and a speed of 150 rpm for 2–3 hr, which allowed complete solubilization of the tissues. The samples were allowed to cool to room temperature; 0.2 mL 30% H_2_O_2_/mL of solution was added to the samples in 0.1 mL aliquots, with tubes swirled between additions. For blood samples, 0.1-mL of 0.1 M EDTA/mL of Solvable was added before 0.3 mL 30% H_2_O_2_/mL of Solvable. After standing at room temperature for 15–30 min, the vials were capped tightly and then heated for another 1 hr in a shaking incubator at 60°C and a speed of 200 rpm. The Solvable, EDTA, and/or H_2_O_2_ alone (at the proportions added to the tissues) were put in another vial and taken through all the heating procedure to correct for background counts. Scintillation fluid (5 mL) was added to 200 μL aliquots of the solubilized tissues, vortexed, and allowed to stand in the dark overnight before counting. For calculating the percent of the dose in the blood, we used a blood volume of 6% of body weight.

## Results

### Dose-dependent stimulation of urinary MeHg excretion by increasing doses of NAC

**Figure 1:**
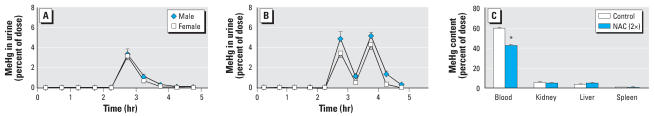
Effect of NAC on urinary MeHg excretion in male and female rats. Animals received [^14^C]MeHg (0.1 μmol/kg body weight) at time zero and NAC (1 mmol/kg body weight) after 2 hr (*A,B*); some animals (*B*) received a second dose of NAC 1 hr later. (*C*) Residual levels of [^14^C]MeHg in selected organs at the end of experiments. Values are mean ± SD; *n* = 4 rats per group. *Significantly different from control (*p* < 0.05).

In agreement with recent findings from our laboratory (Madejczyk et al. 2007), NAC produced a rapid, transient increase in urinary MeHg excretion in rats that had received 0.1 μmol/kg [^14^C]MeHg 2 hr prior to NAC administration (Figure 1). Approximately 5% of the MeHg dose was excreted during the 2 hr after NAC administration in both male and female rats, whereas control animals excreted < 0.1% of the dose over the same time interval (data not shown). Animals that received double doses of NAC had two peaks of excretion, and there were minimal differences between the sexes (Figure 1B). The total percentage excreted in the 2 hr after NAC injection was not different between sexes when a single dose was given, but this became significant when two doses of NAC were given within the same time period, with the male group being slightly higher (Figure 1B). MeHg distributes within tissue compartments at a very rapid rate compared with its rate of excretion, and its elimination follows first-order kinetics (Clarkson 1993). Because MeHg was given iv in these studies, MeHg concentrations in most tissues approached steady-state levels within about 1 hr, and were not subject to fluctuations due to normal excretion at this short time interval. Being a single compartment distribution, the blood levels of MeHg reflect levels in different tissues at constant ratios.

We compared the residual MeHg in selected tissues of the male group that received double doses of NAC with control animals without NAC treatment (Figure 1C). Similar results were obtained for female rats (data not shown). The MeHg was undetectable in the brain of both treated and untreated animals within the short time frame of this experiment; however, the blood levels of MeHg in NAC-treated animals were lower than those in the untreated group (Figure 1C). We observed no differences in the residual MeHg in the liver, kidney, and spleen of the two groups.

**Figure 2:**
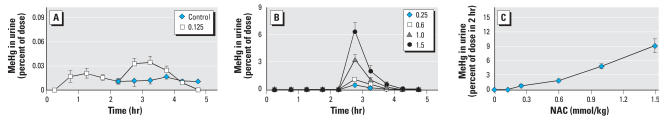
Dose-dependent effects of NAC on urinary MeHg excretion in male rats that received [^14^C]MeHg (0.1 μmol/kg body weight). Two hours after [^14^C]MeHg administration, animals received vehicle or different doses of NAC [vehicle or 0.125 mmol/kg NAC (*A*); 0.25–1.5 mmol/kg NAC (*B*)]. (*C*) Total amounts of [^14^C]MeHg excreted in urine during the 2 hr after NAC injection plotted against the various doses of NAC. Values are mean ± SD; *n* = 4–5 rats per group.

To determine whether the effects of NAC are dose dependent, we treated animals with MeHg at 0.1 μmol/kg and then with different doses of NAC (0.125–1.5 mmol/kg). Control animals excreted only minimal amounts of MeHg in urine (< 0.1% in 2 hr following vehicle injection), but this was markedly enhanced by increasing doses of NAC (Figure 2 A,B). At the lowest NAC dose (0.125 mmol/kg), urinary MeHg excretion was double that of controls during the 2 hr after NAC administration (Figure 2A). At the highest NAC dose tested (1.5 mmol/kg), about 10% of the MeHg dose was excreted in urine in 2 hr (Figure 2B). When the correlation between NAC dose and urinary MeHg excretion was plotted, we observed a nearly linear relationship (Figure 2C).

### A standard dose of NAC stimulates urinary excretion of MeHg with a relatively constant predictive ratio

**Figure 3:**
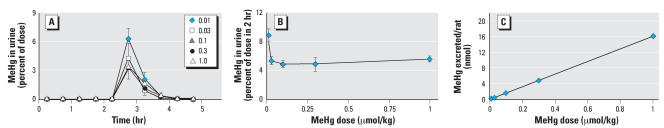
Effect of a standard dose of NAC (1 mmol/kg) on urinary excretion of [^14^C]MeHg. (*A*) Effect of NAC after treatment with various doses of [^14^C]MeHg (μmol/kg) over time. (*B*) Amount of [^14^C]MeHg excreted in urine 2 hr after NAC injection plotted against [^14^C]MeHg doses. (*C*) Actual amount of [^14^C]MeHg excreted in urine versus the amount injected; *y* = 16.133*x* + 0.0281; *R*^2^ = 0.9998. Values are mean ± SD; *n* = 4–5 rats in each group.

To test the hypothesis that a standardized dose of NAC will produce an increase in urinary MeHg excretion that is proportional to the body burden of MeHg, we treated animals with different doses of MeHg (0.01–1.0 μmol/kg), but all groups received a standard dose of 1.0 mmol/kg NAC at 2 hr after MeHg administration. The injection of NAC was followed by a sharp increase in urinary MeHg excretion at all MeHg doses (Figure 3A). Except for the lowest MeHg dose, the percentage excreted in the 2 hr following NAC injection was relatively constant, with a mean ± SD of 5.2 ± 0.3% of dose excreted over this time period (Figure 3B). When the amount of MeHg excreted in urine was plotted against the MeHg dose, we observed a linear relation (Figure 3C).

### NAC in drinking water lowers the body burden and accelerates the urinary excretion of MeHg in pregnant rats

**Figure 4:**
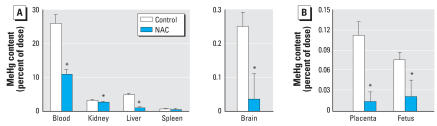
Effect of NAC on body burden and transplacental transfer of MeHg in pregnant rats. Residual levels of [^14^C]MeHg in selected organs (*A*) and in placentas and fetuses (*B*) from pregnant rats that received [^14^C]MeHg (0.1 μmol/kg) via lateral vein on GD14. After 24 hr, NAC-treated rats received 10 mg/mL NAC in their drinking water for 2 days. Values are mean ± SD; *n* = 4 rats in each group or 8 fetoplacental units from 4 rats in each group. *Significantly different from control (*p* < 0.05).

Because fetuses are exposed to MeHg via maternal blood and hence are prone to the danger of developmental abnormalities (Ornaghi et al. 1993), we determined the effectiveness of NAC in reducing the body burden of MeHg in pregnant dams. Pregnant dams were injected with MeHg via the lateral tail vein on GD14; 24 hr later some animals were supplied drinking water containing 10 mg/mL of NAC *ad libitum* for another 48 hr. Animals were then anesthetized, and we removed from each dam two fetoplacental units along with blood, liver, kidney, spleen, and brain for MeHg determination. The residual MeHg was significantly lower in the tissues isolated from the dams exposed to NAC in drinking water than in tissues from untreated dams, including the placenta and fetus (Figure 4A,B). NAC had different effects on individual tissue MeHg levels: blood and liver levels were decreased by approximately 60–80%, whereas kidney MeHg decreased by only 20%. In contrast, MeHg levels in the fetus and in placenta and maternal brain were decreased by approximately 70–90%.

### NAC fails to stimulate urinary MeHg excretion in preweanling rats

**Figure 5:**
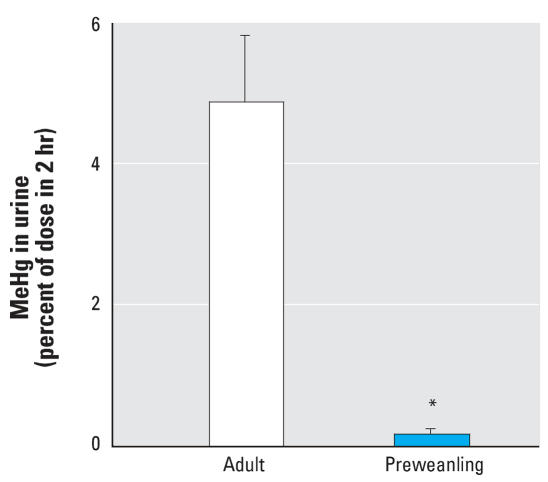
Effects of NAC on urinary MeHg excretion in preweanling and adult rats treated with 0.1 μmol/kg [^14^C]MeHg 1 hr before treatment with 1 mmol/kg NAC. Values shown are amounts of [^14^C]MeHg excreted in urine 2 hr after NAC injection (mean ± SD); *n* = 4–5 rats per group. *Significantly different from adult animals (*p* < 0.05).

Infants whose brains are still developing are at higher risk of MeHg poisoning (Clarkson 2002). Thus, it is important to determine whether NAC is effective in stimulating urinary excretion of MeHg in young animals. To test this possibility, we treated rats between 15–19 days of age with 0.1 μmol/kg MeHg iv, followed by a single dose of NAC 1 hr after MeHg injection. However, NAC administration had only minimal effects on urinary excretion of MeHg in the preweanling animals (Figure 5).

## Discussion

Previous studies from our laboratory have suggested that NAC may be an ideal agent for enhancing MeHg excretion in exposed individuals because of its ability to markedly stimulate MeHg excretion when given orally, its relatively low toxicity, and its wide availability in the clinical setting (Ballatori et al. 1998). The present study provides strong support for this hypothesis. The efficacy of NAC was demonstrated in rats of both sexes, and in a dose-dependent manner. Moreover, NAC was capable of reducing MeHg levels in the fetus, indicating that NAC may be protective against MeHg embryotoxicity. In contrast, NAC was ineffective when administered iv to preweanling rats; this suggests that the transport mechanisms responsible for NAC stimulation of MeHg excretion are not yet mature in these animals. In addition, our results demonstrate that the amount of MeHg excreted into urine after NAC challenge is proportional to the MeHg body burden, indicating that NAC may be useful as a bio-monitoring agent.

The increase in urinary MeHg excretion was directly dependent on the dose of NAC. By administering 1 mmol NAC/kg to male and female rats exposed to 0.1 μmol MeHg, about 5% of the body burden was excreted in urine in < 2 hr; this effect doubled within the same time period when two doses of NAC were applied at 1-hr intervals. At double doses of NAC, differences in the percentage of excreted MeHg between the sexes became apparent, being somewhat higher in male than in female rats (Figure 1B). Although the reason for this difference is unknown, it may be related to differences in the expression of transporters that are thought to be involved in the transport of the MeHg–NAC complex, or of NAC itself, across the renal tubular cells (Madejczyk et al. 2007). Sex is known to influence pharmacokinetic parameters such as clearance and half-life of many drugs (Buist et al. 2002). For example, the expression of the basolateral membrane organic anion transporter-1 (*Oat1*), which has been implicated in the transport of MeHg–NAC complex (Koh et al. 2002), is greater in male rats than in females (Buist et al. 2002).

In the present study we also found a dramatic reduction in body burden of MeHg when NAC was administered to pregnant rats via the drinking water (Figure 4A). Also, the placental and fetal MeHg levels of the pregnant rats were significantly reduced (Figure 4B). These observations further suggest that NAC may be an excellent agent for enhancing MeHg elimination in exposed individuals. Blood and liver levels of MeHg were decreased by 60–85%, and comparable decreases were seen in crucial tissues such as the brain (from 0.3% of the dose to 0.03%; 90% decrease), placenta (from 0.1% to 0.01%; 90% decrease), and fetus (0.08% to 0.02%; 75% decrease). Thus, in addition to being an antioxidant, which has been attributed to its protective role against MeHg embryotoxicity (Ornaghi et al. 1993), the present study demonstrates that NAC actually lowered MeHg levels in fetuses and placenta.

Our findings therefore indicate that NAC may be very useful in the therapeutic management of pregnant women whose babies are in danger of prenatal MeHg poisoning. Mass health disasters in Minamata and Niigata, Japan, and in Iraq have confirmed that MeHg is neurotoxic and that the prenatal period is the most sensitive stage of the life cycle (Davidson et al. 1998). Thus, even though controversies may surround the maternal levels of MeHg that predispose to future neurologic problems in children (Davidson et al. 1998; Grandjean et al. 1997), it is prudent to decrease MeHg levels, even in asymptomatic women living in areas with a history of dependence on seafoods that are highly contaminated with MeHg. Because the NAC doses used in the present study are comparable with those used in humans who have overdosed on acetaminophen (i.e., 140 mg/kg or 0.86 mmol/kg) (Smilkstein et al. 1991; Woo et al. 2000), we speculate that a similar NAC dosing regimen as used in aceta-minophen overdoses would likely be safe and effective in accelerating MeHg excretion in humans. Limited data based mainly on case reports of treatment of acetaminophen overdose in pregnancy suggest that NAC may also be safely administered during pregnancy (Wilkes et al. 2005). Thus, NAC may be a safe therapeutic agent in pregnant women to decrease the levels of this toxic agent in developing embryos.

Interestingly, NAC failed to significantly increase the urinary excretion of MeHg in pre-weanling rats (postnatal days 15–19; Figure 5). This may not be surprising because age is known to influence pharmacokinetic parameters, including the extent of and sensitivity to drug effects (Fanos and Dall’Agnola 1999; Tune 1975). Moreover, the expression levels of transporters that are thought to be involved in the transport of the MeHg–NAC complex across the renal tubule cells are immature in preweanling rats. In particular, expression of *Oat1* in rat kidney is low at birth, but it approaches adult levels at around 30 days of age (Buist et al. 2002). Oat3 in the kidney of developing rats is expressed as early as postnatal day 10 (Buist et al. 2002); however, Oat3 does not seem to participate in the transport of the MeHg–NAC complex (Koh et al. 2002). Likewise, the expression of the apical membrane organic anion transporter Mrp2 (multi-drug resistance-associated protein-2), which is thought to participate in the tubular transport of MeHg–NAC (Madejczyk et al. 2007), is also low in the preweanling animal (Maher et al. 2005; Tomer et al. 2003) and may contribute to the inability of NAC to increase MeHg excretion in young animals. Thus, the present findings support the involvement of *Oat1* and *Mrp2* in the urinary excretion of the MeHg–NAC complex (Koh et al. 2002; Madejczyk et al. 2007) and conform to the reports on developmental expression of transporters (Buist et al. 2002). In contrast, our results also indicate that NAC may not be useful as a complexing agent in one age group that is relatively vulnerable to the neurotoxic effect of MeHg—neonatal animals.

**Figure 6:**
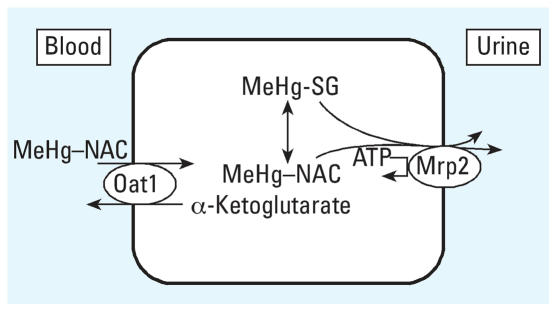
Potential mechanism of NAC-stimulated renal excretion of MeHg. The MeHg–NAC complex spontaneously formed in the blood is a substrate for Oat1, an α-ketoglutarate–coupled anion exchanger at the basolateral membrane of proximal tubule cells. Once inside the cell, some of the MeHg will redistribute to other intracellular ligands, including the formation of the glutathione complex (MeHg–SG). The MeHg–NAC and MeHg–SG complexes are both substrates for Mrp2, an ATP-dependent transporter localized to the brush border membrane, which mediates efflux of these complexes from the renal tubular cells into the renal tubular lumen for excretion via the urine. Modified from Madejczyk et al. (2007).

Figure 6 illustrates a model that may explain the effects of NAC on urinary MeHg excretion. After oral NAC administration, NAC is rapidly absorbed from the gastrointestinal tract, and blood NAC levels rise quickly (Borgstrom et al. 1986; Rodenstein et al. 1978). NAC can spontaneously (i.e., nonenzymatically) form a thermodynamically stable mercaptide complex with MeHg to form MeHg–NAC. MeHg–NAC is an excellent substrate for Oat1 (Koh et al. 2002), a major renal basolateral membrane organic anion carrier, and thus MeHg–NAC can be transported from blood into the renal tubular cell via this carrier. It is important to note that NAC itself is also efficiently cleared by the kidney and excreted into urine in high concentrations (Borgstrom et al. 1986; Rodenstein et al. 1978). In humans the half-life of NAC in blood plasma is only 2 hr; this short half-life is due largely to NAC’s rapid urinary excretion (Borgstrom et al. 1986; Rodenstein et al. 1978). Approximately one-third of the NAC is excreted in urine during the first 12 hr after administration (Borgstrom et al. 1986); this half-life for NAC in blood is also consistent with the rapid acceleration of MeHg excretion observed during NAC administration, and with the rapid deceleration in MeHg excretion after NAC withdrawal (Figures 1–3).

Once the MeHg–NAC complex enters the renal tubular cell, MeHg may exchange with other thiols, including reduced glutathione (GSH), to form MeHg–SG (Figure 6), although a significant fraction likely remains as MeHg–NAC, given the high amount of NAC present under these conditions. Both the NAC and GSH complexes are substrates for the apically located, ATP-driven Mrp2 transport protein (Ballatori 2002; Madejczyk et al. 2007), thus providing an efficient mechanism for excretion of MeHg into renal tubular fluid for eventual excretion in urine (Figure 6).

Findings of the present study also demonstrate that NAC offers biomonitoring potential for exposure to MeHg. The results from rats exposed to a wide range of MeHg doses showed that NAC at an iv dose of 1 mmol/kg body weight produced a urinary excretion equivalent to about 5% of the body burden in < 2 hr (Figure 3B). Although the latter dose–response studies with NAC were performed using iv administration, the observation that NAC in drinking water (oral dosing) resulted in the excretion of about 4% of the dose in the first 24 hr (Madejczyk et al. 2007) and the fact that NAC is highly effective at enhancing urinary MeHg excretion when given either orally or iv (Ballatori et al. 1998; Madejczyk et al. 2007) make it highly likely that it will also be an effective biomonitoring agent when given orally. However, additional studies are needed to test this hypothesis and to examine whether NAC is also effective in humans exposed to MeHg. Nevertheless, the findings that NAC is both relatively selective for MeHg and quick-acting are remarkable, and also suggest that an oral NAC challenge test may be useful for monitoring MeHg body burden. As noted previously (Boischio and Cernichiari 1998; Boischio et al. 2000; Cox et al. 1995), although hair is an excellent biomarker of MeHg exposure, hair growth rate is only about 1 cm/month. Therefore, the hair level of MeHg may not provide an early warning before the onset of neurologic effects after an acute exposure to MeHg. A safe chelation challenge is thus a preferable choice for preventive purposes because it reflects the status of the body burden at a particular point in time. Thus, if a person has an elevated body burden of MeHg, the administration of NAC is expected to cause a short-term increase in its urinary excretion (Frumkin et al. 2001).

Because MeHg is less toxic to the kidney compared with inorganic mercury (Aleo et al. 2005; Lash et al. 2005), the rapid elimination of MeHg via urine after NAC administration should be a safe therapeutic and diagnostic option. Rapid urinary excretion also ensures that MeHg is quickly eliminated before it is significantly demethylated to inorganic mercury, a form that is more nephrotoxic. Moreover, in contrast to other complexing agents (Aposhian et al. 1995; Cantilena and Klaassen 1982), NAC does not alter tissue distribution of essential metals (Hjortso et al. 1990). Because of its nucleophilic properties, NAC is also able to inactivate electrophiles and free radicals directly through conjugation and reduction (Moldeus et al. 1986). More importantly, findings of the present study show that NAC is effective at enhancing MeHg excretion when given either orally or iv. In addition, NAC is a powerful antioxidant and is widely available in clinical settings, where it is being used both orally and iv at a dose of 140 mg/kg for treating acetaminophen (paracetamol) toxicity (Smilkstein et al. 1991; Woo et al. 2000); this dose of NAC is comparable to the dose we used in the present study. Thus, NAC may be effective at enhancing MeHg excretion in exposed individuals, and it should be evaluated for both biomonitoring purposes and for decreasing the MeHg body burden in humans.
